# Changes in Muscle Activity Patterns and Joint Kinematics During Gait in Hemophilic Arthropathy

**DOI:** 10.3389/fphys.2019.01575

**Published:** 2020-01-31

**Authors:** Carlos Cruz-Montecinos, Sofía Pérez-Alenda, Felipe Querol, Mauricio Cerda, Huub Maas

**Affiliations:** ^1^Department of Physiotherapy, University of Valencia, Valencia, Spain; ^2^Department of Human Movement Sciences, Faculty of Behavioural and Movement Sciences, Amsterdam Movement Sciences, Vrije Universiteit Amsterdam, Amsterdam, Netherlands; ^3^Laboratory of Clinical Biomechanics, Department of Physical Therapy, Faculty of Medicine, Universidad de Chile, Santiago, Chile; ^4^SCIAN-Lab, Anatomy and Developmental Biology Program, Faculty of Medicine, Institute of Biomedical Sciences, Universidad de Chile, Santiago, Chile; ^5^Biomedical Neuroscience Institute, Santiago, Chile

**Keywords:** hemophilic arthropathy, electromyography, knee joint, ankle joint, gait analysis, lower limb kinematics, joint damage

## Abstract

Hemophilic arthropathy is the result of repetitive intra-articular bleeding and synovial inflammation. In people with hemophilic arthropathy (PWHA), very little is known about the neural control of individual muscles during movement. The aim of the present study was to assess if the neural control of individual muscles and coordination between antagonistic muscle pairs and joint kinematics during gait are affected in PWHA. Thirteen control subjects (CG) walked overground at their preferred and slow velocity (1 m/s), and 14 PWHA walked overground at the preferred velocity (1 m/s). Joint kinematics and temporal gait parameters were assessed using four inertial sensors. Surface electromyography (EMG) was collected from gluteus maximus (GMAX), gluteus medius (GMED), vastus medialis (VM), vastus lateralis (VL), rectus femoris (RF), medial gastrocnemius (MG), lateral gastrocnemius (LG), soleus (SOL), tibialis anterior (TA), semitendinosus (ST), and biceps femoris (BF). Waveforms were compared using the time-series analysis through statistical parametric mapping. In PWHA compared to CG, EMG amplitude during the stance phase was higher for LG (for both velocities of the CG), BF (slow velocity only), and ST (preferred velocity only) (*p* < 0.05). Co-contraction during the stance phase was higher for MG-TA, LG-TA, VL-BF, VM-ST, LG-VL, and MG-VM (both velocities) (*p* < 0.05). MG and LG were excited earlier (preferred velocity only) (*p* < 0.05). A later offset during the stance phase was found for VL, BF, and ST (both velocities), and BF and GMAX (preferred velocity only) (*p* < 0.05). In addition, the range of motion in knee and ankle joints was lower in PWHA (both velocities) and hip joint (preferred velocity only) (*p* < 0.05). In conclusion, the neural control of individual muscles and coordination between antagonistic muscles during gait in PWHA differs substantially from control subjects.

## Introduction

Hemophilia is an X chromosome-linked bleeding disorder caused by a deficiency of coagulation factors VIII (hemophilia A) and factor IX (hemophilia B) (Oldenburg et al., 2004). The prevalence of hemophilia A (12.8 per 100,000 male) is higher than that of hemophilia B (1.6 per 100,000 male) (Stonebraker et al., 2010, 2012). The most common clinical manifestation of hemophilia is arthropathy, affecting 90% of people with severe hemophilia (Manco-Johnson et al., 2004). Hemophilic arthropathy is the result of repetitive intra-articular bleeding and synovial inflammation, characterized by joint impairment, chronic pain, and reduced quality of life (Fischer et al., 2005, 2016; Krüger et al., 2018; Roussel, 2018). The intra-articular bleeding and the inflamed synovium generate an irreversible change in cartilage tissue (Pulles et al., 2017). This is mediated by chondrocyte apoptosis, resulting in the inability of chondrocytes to restore proteoglycan synthesis, eventually leading to joint destruction (Hooiveld et al., 2003). The synovial hypertrophy and hypervascularization increase the sensitivity for bleedings during tasks involving low loads on the joints (Melchiorre et al., 2017; Pulles et al., 2017). Animal studies have reported that a single hemarthrosis results in irreversible damage of cartilage (Madhok et al., 1988; Roosendaal et al., 1997; Hooiveld et al., 2003; Hakobyan et al., 2008), indicating that joint impairment in people with hemophilic arthropathy (PWHA) could be observed following a single hemarthrosis event.

In the lower limb of PWHA, the knee and ankle joints are the most commonly affected. The joint damage is accompanied by changes in the properties of the neuromusculoskeletal system, as a reduced passive range of joint motion (Soucie et al., 2004), muscle size (Stephensen et al., 2012), maximal muscle force (Hilberg et al., 2001; González et al., 2007), tendon stiffness (Cruz-Montecinos et al., 2019b), and impaired proprioception (Hilberg et al., 2001). In addition, static postural control and lower limb kinematics during gait are affected in PWHA (Gallach et al., 2008; Lobet et al., 2010, 2012; Stephensen et al., 2012; Cruz-Montecinos et al., 2017; García-Massó et al., 2019). However, we know little about the consequences of these changes on neuromuscular control of gait. Applying a muscle synergy approach (Tresch et al., 2006; Steele et al., 2015), it was found that, during gait in PWHA compared to healthy controls, the total variance of electromyography (EMG) accounted for by one muscle synergy was higher (Cruz-Montecinos et al., 2019a). This result suggests increased co-contraction of antagonistic muscles. However, the muscle synergy analysis does not yield information about potential differences in the neural control of individual muscles and changes in co-contraction between antagonistic muscle pairs (De Groote et al., 2014).

The coordination between antagonistic muscles during dynamic activities such as gait is key in the understanding of the progression of joint degeneration. In knee osteoarthritis (OA), the co-contraction between knee flexors extensors during the stance phase has been related to the progression of knee OA and greater cartilage loss (Hubley-Kozey et al., 2009; Hodges et al., 2016). A higher co-contraction between superficial ankle plantar flexors and dorsiflexors during the stance phase has been also reported in people with ankle OA (Doets et al., 2007; Von Tscharner and Valderrabano, 2010). Similar changes in co-contraction between muscles crossing knee and ankle joints during gait may be expected in PWHA. A better understanding of the individual muscle activity patterns and co-contraction between antagonists during gait in PWHA may be used to improve rehabilitation strategies aimed at increasing muscle strength (Calatayud et al., 2019), for neuromuscular re-education (Preece et al., 2016), or to improve the feedback strategies during gait (Booth et al., 2019), as well the orthopedic surgeries (Rodriguez-Merchan, 2012).

The aim of the present study was to assess if the neural control of individual muscles, coordination between antagonistic muscle pairs, and joint kinematics during gait are affected in PWHA. For this purpose, EMG acitivity of several leg muscles and joint kinematics were recorded in PWHA and a control group (CG). Waveforms were compared using the time-series analysis through statistical parametric mapping (SPM) (Pataky, 2010).

## Materials and Methods

### Participants

This study was approved by the local ethical committee and conducted in agreement with the Declaration of Helsinki. The data of the present study have been used for a previous paper, which addressed a different research question, using other data analysis methods (i.e., muscle synergies) and focused on different outcome measures (Cruz-Montecinos et al., 2019a). All participants were informed about the purpose and procedures of the project and gave their written informed consent to participate in the study. Fourteen PWHA and 13 healthy control subjects were recruited (for the characteristics of each group, see Table 1).

**TABLE 1:** Clinical characteristics between groups.

**Characteristic between groups**	**CG = 13**	**PWHA = 14**	***p* value**
Age (years)	28.4 ± 6.1	28.4 ± 6.6	0.991
Body mass (kg)	75.5 ± 8.0	73.9 ± 11.6	0.687
Height (cm)	175.6 ± 4	171.7 ± 8	0.115
Body mass index	24.4 ± 1.9	25.0 ± 3.4	0.593
Pain during 30 m walk	0 [0 0]	1 [0 5]	**0.002**
Duration of pain (years)	NA	7.5 [5 20]	NA
Pain medication	NA	4/14	NA
Opioids medication	NA	0/14	NA
Physical activity (>150 min/week)	7/13	4/14	0.345

*People with hemophilic arthropathy (PWHA; *n* = 14). Control group (CG; *n* = 13). Parametric distribution: mean ± SD. Non-parametric distribution: median [range]. NA, not applicable. Significant differences (*p* < 0.05) is in bold.*

#### People With Hemophilic Arthropathy

Inclusion criteria: Males, diagnostic hemophilia A or B severe and moderate, hemophilic arthropathy with a minimum of 1 point (in knee or ankle) of the radiological Pettersson score assessed with X-ray examination, over 18 years of age and under 45 years, passive range of motion (ROM) of the knee >60° and >20° in ankle [both values correspond to approximately 30 and 40% of the normal ROM (Soucie et al., 2011), respectively], prophylaxis treatment with deficient factor (i.e., XIII or IX), and body mass index less than 30. Exclusion criteria: History of hip, knee or ankle arthroplasty, equinus foot, inability to walk without an assistive device (e.g., walker, cane), history of muscle or joint bleeding in lower limbs in the last 2 months, chronic cardiac and/or respiratory pathology and neurological disease.

#### Control Subjects

Inclusion criteria were the following: male, over 18 years of age and under 45 years, no hemophilia, and body mass index lower than 30. Exclusion criteria were the following: a Hemophilia Joint Health Score (see below) > 0, traumatic injuries; signs or symptoms of injury or symptomatic arthritis to the trunk, lower back, and lower limb within the past 3 months; which affects movement or function in the lower limb, any single positive findings of the Alt-man’s criteria for knee OA (i.e., morning stiffness < 30 min, crepitus, bony tenderness, bony enlargement, palpable warmth) (Altman et al., 1986; Na and Buchanan, 2019); history of musculoskeletal surgery in the lower limb and spine; scoliosis; history of acute or chronic musculoskeletal disorders; cardiac and/or respiratory pathology; and neurological disease.

### Surface Electromyography Protocol for the Lower Limb

**FIGURE 1:**
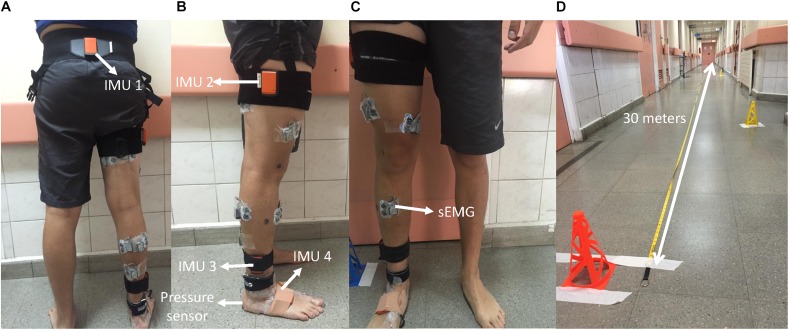
Sensors locations and 30 m used corridor. **(A)** Back view. **(B)** Lateral view. **(C)** Frontal view. **(D)** Walking assessment corridor. Surface electromyography (sEMG), inertial sensor positioned in pelvis (IMU 1), inertial sensor positioned in lateral face of thigh (IMU 2), inertial sensor positioned lateral face of the shank (IMU 3), and inertial sensor positioned in midfoot (IMU 4).

In PWHA, the limb with the highest points on the radiological Pettersson score was selected. In CG, the dominant limb was assessed. Leg dominance was assessed by asking the subjects which leg they would use to kick a ball (Bejarano et al., 2017). After shaving and cleaning the skin with alcohol, surface electrodes (Ag–AgCl, Kendall H124SG) were placed (interelectrode spacing 2 cm) on the following muscles according to SENIAM guidelines (Hermens et al., 2000): medial gastrocnemius (MG), lateral gastrocnemius (LG), soleus (SOL), tibialis anterior (TA), vastus lateralis (VL), medialis (VM), rectus femoris (RF), semitendinosus (ST), biceps femoris (BF), gluteus maximus (GMAX), and gluteus medius (GMED). Activity patterns of these muscles were measured using a wireless EMG system (MyoSystem DTS, Noraxon USA Inc., Scottsdale, CA, United States), with a sampling rate of 1,500 Hz. Heel strike was detected by a synchronized wireless pressure sensor (Noraxon USA Inc., Scottsdale, CA, United States) placed underneath the heel of the foot (Figure 1).

### Kinematics of the Lower Limb

Based on inertial measurement units (IMUs), the sagittal kinematics of the hip, knee, and ankle joints were assessed. Four IMU sensors (Xsens, Enschede, Netherlands) were positioned between posterior superior iliac spines, the lateral face of the thigh within the proximal third, lateral face of the shank within the distal third close to the lateral malleolus, and the midfoot (Cutti et al., 2010). The sensors placed on sacrum, thigh, and shank were fixed with the fixation system provided by the company (Xsens, Enschede, Netherlands). The sensor placed on midfoot was fixed by adhesive elastic taping (Leukotape K, BSN Medical, Hamburg, Germany) with sufficient tension to avoid movement artifacts (Figure 1). The Xsens system and the traditional camera-based optical motion capture systems have reported similar flexion–extension hip, knee, and ankle waveforms (coefficient of multiple correlation > 0.96) for hip, knee, and ankle angle during overground walking, with average angle estimation errors of 2.15, 1.87, and 2.47°, respectively (Zhang et al., 2013). Data were collected at a sampling frequency of 75 Hz. The IMUs and EMG/pressure sensor were synchronized through a trigger pulse.

### Experimental Protocol

Each subject was invited to walk barefoot overground at their preferred velocity and the CG also walked at a slower velocity (1.0 m/s) similar to that of the mean preferred velocity in PWHA. Two velocities were tested for the CG because joint kinematics and muscle activity patterns are dependent on gait velocity (Kirtley et al., 1985; Tirosh and Sparrow, 2005). For the CG, the slow velocity was practiced three times for 10 m. Subsequently, each subject walked for 30 m twice (Figure 1). Mean velocity was assessed by dividing total distance by total time. To reduce the risk of muscular and intramuscular bleeding during the experimental procedures, 2 min of rest in between tests were allowed. The PWHA received prophylactic treatment 1 and 2 h before the experiment.

### Data Analysis

For EMG and kinematic analysis, we used Matlab software 2016 (Statistics and Machine Learning Toolbox, MathWorks, Inc., Natick, MA, United States).

#### EMG Signal Processing

In each subject, a total of 20 cycles were used for the final analysis. First, a bandpass filter (20–500 Hz, Butterworth, fifth order) was applied. The EMG signals during each step cycle were time normalized to 0–100%. To assess EMG amplitudes, the EMG signals were rectified using Hilbert transformation and smoothed with a low-pass filter at 6 Hz (Hubley-Kozey et al., 2006; Rutherford et al., 2011, 2017). The amplitude for each muscle was normalized to the maximum value of all included steps (i.e., 20 cycles) separately for each group and velocity (i.e., CG during preferred velocity, CG during slow velocity, and PWHA during preferred velocity). This procedure indicates at what periods during the gait cycle that the muscle is relatively more active (Benoit et al., 2003; Cronin et al., 2015). With this method, differences between injured and non-injured legs in relative intensity and timing of EMG activity have been reported for several clinical populations (i.e., ankle OA, ankle arthrodesis, and Achilles tendon surgery) (Wu et al., 2000; Doets et al., 2007; Suydam et al., 2015). The normalized EMG signals were used to calculated the co-contraction index (CCI) at each point of the gait cycle, providing a time-series curve to describe the temporal and magnitude components of the EMG signals based on the following formula (Rudolph et al., 2000; Knarr et al., 2012),

$${\text{CCI}}_{i}=\frac{{\text{LEMG}}_{i}}{{\text{HEMG}}_{i}}\,\left({\text{LEMG}}_{i}+{\text{HEMG}}_{i}\right),$$

where *i* is the point of the gait cycle, LEMG is the normalized magnitude of the EMG for the less active muscle, and HEMG is the normalized magnitude of the EMG for the most active muscle (Knarr et al., 2012). The CCI was calculated for the following antagonistic muscle pairs crossing the ankle (MG-TA, LG-TA, and SOL-TA) and knee joint (VL-BF, VM-ST, LG-VL, and MG-VM). CCI goes from 0 to 2, where a CCI value of 2 indicates the maximum normalized value for both LEMG and HEMG.

The EMG bursts were identified using the *k*-means cluster analysis applied on the bandpass filter and rectified signal using Hilbert transformation. Three clusters were assigned to *k*-means cluster analysis, where the lowest cluster reflects inactivity (Den Otter et al., 2006; Bernabei et al., 2017). Then, EMG burst on- and offset were identified, using the following criteria: every burst shorter than 5 ms was discarded; bursts separated by <125 ms were considered the same burst (Merlo et al., 2003; De La Fuente et al., 2018). For the final analysis, the mean of the 20 cycles was used to represent the muscle temporal and intensity patterns, co-contraction, and the on-/offset of individual muscles for each subject.

#### Flexion–Extension Angle Estimation and Temporal–Spatial Parameter During Gait

To define the sensor to segment alignment, subjects were asked to stand still in a neutral position with their feet parallel, one-foot width apart, and legs and back straight for 10 s (Laudanski et al., 2013). Each axis was reset to define (*x*, *y*, *z*) coordinate. Based on recommendations of the International Society of Biomechanics, the *x*-axis was defined as the anteroposterior axis, *y*-axis as the vertical axis, and *z*-axis as the mediolateral axis (Wu et al., 2002).

The Euler angles of each IMU were determined using the *quat2angle* function in Matlab Software (version 2016; MathWorks, Inc., Natick, MA, United States) using as input the IMU quaternion orientation. To identify the flexion–extension plane, a sequence of bipodal independent flexions of trunk, hip, knee, and ankle were used as reference before starting the protocol. The hip, knee, and ankle angles were defined as flexion–extension angles of the distal body segment with respect to the proximal body segments (Laudanski et al., 2013). Low-pass Butterworth filters were applied to the IMU joint angle data, with a cutoff frequency of 10 Hz. The segmentation of kinematics signals were determined through gait events signifying heel strike and toe-off from the angular velocity in *z*-axis obtained from the IMU sensor of the shank (De Vroey et al., 2018). This method has a strong agreement and excellent correlations with temporal parameters of gait evaluated by means of a camera-based motion capture system (De Vroey et al., 2018). The kinematic signals during each step were normalized to total stride time determined with IMU sensor of the shank. For the final analysis, the mean of the same 20 cycles selected for the EMG analysis were used to represent the kinematics and temporal parameters of gait. To assess the total time of the 30-m walking test, the acceleration of the IMU sensor of the shank was used.

### Clinical Assessments for PWHA

To assess the intensity of pain (scale 0–10 points) during barefoot walking, the Visual Analogue Scale was used. A physical therapist with 10 years of experience in hemophilia rehabilitation (CCM) applied the Hemophilia Joint Health Score 2.1 (HJHS) (Sun et al., 2014; Gouw et al., 2019). The HJHS 2.1 score is used to assess the health status of the joints most commonly affected by bleeding in hemophilia: the knees, ankles, and elbows. This scale consists of eight items per joint (scale 0–20), evaluating (1) joint swelling (0–3 points), (2) duration of swelling (0–1 pts), (3) muscle atrophy (0–2), (4) strength (0–4), (5) crepitus on motion (0–2 points), (6) flexion loss (0–3 points), (7) extension loss (0–3 points), and (8) pain (0–2 points) (Sun et al., 2014). The radiological Pettersson score (scale 0–13) (Pipe and Valentino, 2007) was assessed by a medical doctor (FQ) with more than 30 years of experience in hemophilia.

### Statistical Analysis

*A priori* power analysis conducted in G^∗^Power (3.1.9.2 version) software (Heinrich-Heine-Universität Düsseldorf, Düsseldorf, Germany) showed that 13 subjects per group this design were sufficient to obtain a statistical power of 0.80 at a large effect size (Cohen’s *d* = 1.03; Cruz-Montecinos et al., 2019a), with an alpha = 0.05.

For all statistical analysis, we used Matlab 2016 (MathWorks, Inc., Natick, MA, United States). The normality of data was evaluated through the Shapiro–Wilk test. For all comparison, the alpha level was set at 0.05. To compare the clinical characteristics between groups, the independent samples *t* test was used for age, body mass, height, and body mass index. The Wilcoxon rank-sum test and the chi-squared test was used for pain during walking and physical activity (>150 min/week). Data are expressed as the mean ± standard deviation for normal distribution and median (range) for no-normal distribution.

Two independent assessments were made for all variables: (a) the comparison of CG during preferred walking velocity (CG−pref) with PWHA and (b) and a comparison of CG during slow velocity (CG−slow) with PWHA.

To test for differences between groups in EMG timing amplitude, co-contraction, and kinematics of hip, knee, and ankle, the time-series statistical analysis was applied using the MATLAB-based spm1d-package for *n*-dimensional SPM^1^ (Pataky, 2010). The group comparisons were then carried out using two-tailed independent samples *t* test. The EMG and kinematics time series were considered significantly different if any values of SPM over the entire gait cycle exceeded the critical threshold (alpha = 0.05). In the final step, cluster-specific *p* values were calculated over the entire gait cycle (Pataky, 2010). Cohen’s *d* effect was calculated when statistically significant differences were observed. The mean value of Cohen’s *d* over to the critical threshold of SPM analysis was considered as representative value of effect size.

For the on–off burst detection and temporal gait parameters, the two-tailed independent samples *t* test was used. To determine the effect sizes for the temporal gait parameters and on–off burst detection, the Cohen’s *d* was calculated (*d*: small ≥ 0.2, medium ≥ 0.5, and large ≥ 0.8).

## Results

### Clinical Characteristics

All PWHA were diagnosed with hemophilia A (11 severe and 3 moderate) (for characteristics between groups, see Table 1). The joint damage in PWHA showed similar values of the clinical and radiological status for the knee and ankle joint (see Table 2).

**TABLE 2:** Clinical joint assessment.

**Clinical joint assessment in PWHA**	**Joint**	**Points**
Hemophilia Joint Health Score 2.1 (0–20 points)	Ankle	7.1 ± 3.4
	Knee	6.1 ± 5.2
Radiological Pettersson score (0–13 points)	Ankle	5.9 ± 4.1
	Knee	3.4 ± 3.4

*Joint assessment for people with hemophilic arthropathy (PWHA; *n* = 14). Data are expressed as mean and standard deviation.*

### Muscle Activity Patterns and Co-contraction

**FIGURE 2:**
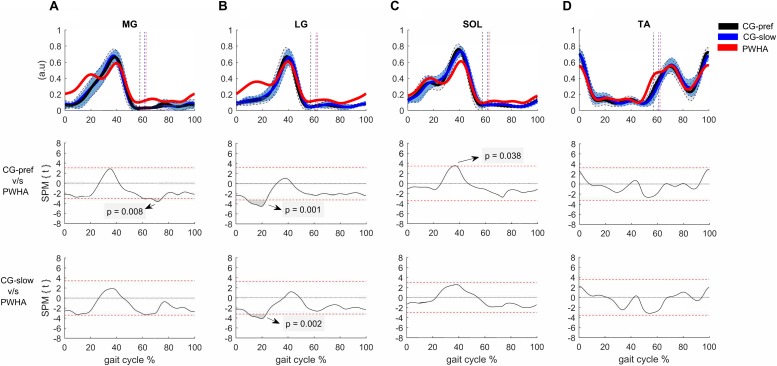
Comparison between groups of surface electromyography of muscles crossing the ankle. **(A)** Medial gastrocnemius (MG). **(B)** Lateral gastrocnemius (LG). **(C)** Soleus (SOL). **(D)** Tibialis anterior (TA). **(Top row**) Surface electromyography of muscles crossing the ankle of people with hemophilic arthropathy (PWHA; *n* = 14) at their preferred velocity (red), the control group (*n* = 13) at their preferred velocity (CG-pref, black), and during the slow velocity condition (CG-slow, blue). Data are plotted as a function of normalized step time (0, heel strike) and expressed as mean with 95% confidence interval for CG. Vertical dashed lines indicate transition from stance to swing phase (black, CG-pref; blue, CG-slow; red, PWHA). **(Bottom two rows)** Time-dependent *t* values of the statistical parametric mapping (SPM) for groups comparison. Horizontal red dashed line indicates *p* = 0.05 level. Gray zones indicate regions with statistically significant differences. a.u, Arbitrary unit.

**FIGURE 3:**
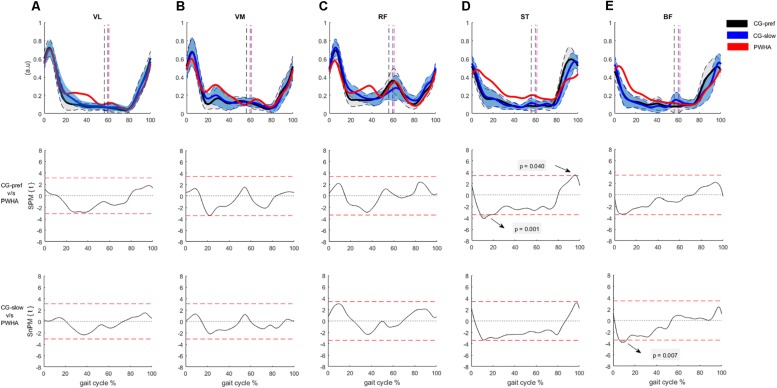
Comparison between groups of surface electromyography of muscles crossing the knee. **(A)** Vastus lateralis (VL). **(B)** Vastus medialis (VM). **(C)** Rectus femoris (RF). **(D)** Semitendinosus (ST). **(E)** Biceps femoris (BF). **(Top row)** Surface electromyography of muscles crossing the knee of people with hemophilic arthropathy (PWHA; *n* = 14) at their preferred velocity (red), the control group (*n* = 13) at their preferred velocity (CG-pref, black), and during the slow velocity condition (CG-slow, blue). Data are plotted as a function of normalized step time (0, heel strike) and expressed as mean with 95% confidence interval for CG. Vertical dashed lines indicate transition from stance to swing phase (black, CG-pref; blue, CG-slow; red, PWHA). **(Bottom two rows)** Time-dependent *t* values of the statistical parametric mapping (SPM) for groups comparison. Horizontal red dashed line indicates *p* = 0.05 level. Gray zones indicate regions with statistically significant differences. a.u, Arbitrary unit.

**FIGURE 4:**
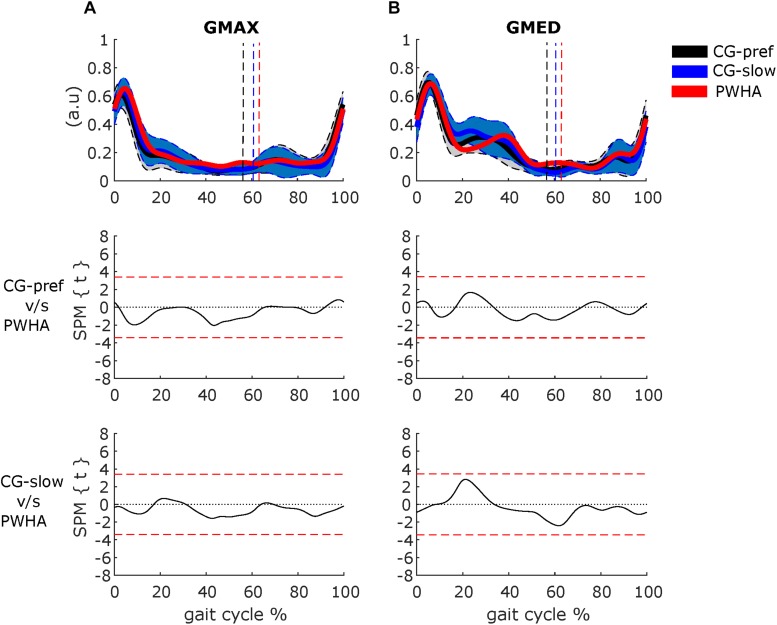
Comparison between groups of surface electromyography of muscles crossing the hip. **(A)** Gluteus maximus (GMAX). **(B)** Gluteus medius (GMED). **(Top row)** Surface electromyography of muscles crossing the hip of people with hemophilic arthropathy (PWHA; *n* = 14) at their preferred velocity (red), the control group (*n* = 13) at their preferred velocity (CG-pref, black), and during the slow velocity condition (CG-slow, blue). Data are plotted as a function of normalized step time (0, heel strike) and expressed as mean with 95% confidence interval for CG. Vertical dashed lines indicate transition from stance to swing phase (black, CG-pref; blue, CG-slow; red, PWHA). **(Bottom two rows)** Time-dependent *t* values of the statistical parametric mapping (SPM) for groups comparison. Horizontal red dashed line indicates *p* = 0.05 level. Gray zones indicate regions with statistically significant differences. a.u, Arbitrary unit.

Comparing the muscle activity patterns between groups at their preferred velocity revealed different activity patterns in the ankle plantar flexors and knee flexors. In the muscles crossing the ankle, PWHA showed a relatively higher activation of MG during the swing phase (at ∼70% of the gait cycle; *p* = 0.008, *d* = 1.26), a relatively higher activation of LG during the stance phase (at around 10–20% of the gait cycle; *p* = 0.001, *d* = 1.56) (Figure 2), and a relatively lower activation of SOL during the propulsion phase (at ∼40% of the gait cycle; *p* = 0.038, *d* = 1.35). In the muscles crossing the knee, PWHA showed a relatively higher activation of ST during the stance phase (at ∼10% of the step cycle; *p* = 0.001, *d* = 1.39) and lower at the end of the swing phase (at ∼95% of the gait cycle; *p* = 0.040, *d* = 1.31) (Figure 3). For the muscles crossing the hip, no differences between group were found for GMAX and GMED (Figure 4).

**FIGURE 5:**
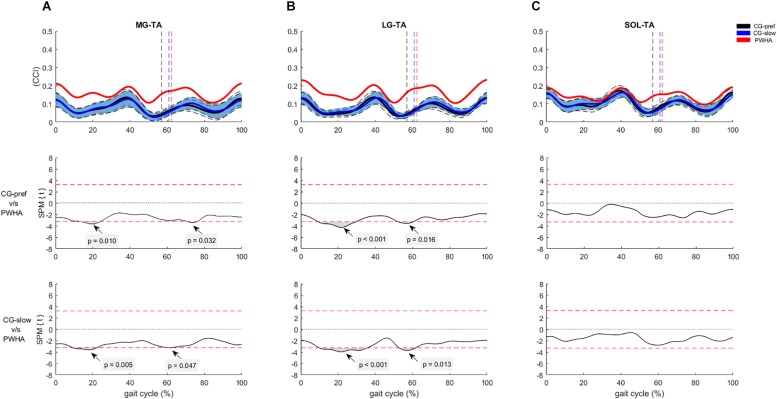
Co-contraction of muscles crossing the ankle during gait. **(A)** Con-contraction index (CCI) between medial gastrocnemius and tibialis anterior (MG-TA). **(B)** CCI between lateral gastrocnemius and tibialis anterior (LG-TA). **(C)** CCI between soleus and tibialis anterior (SOL-TA). **(Top row)** CCI of muscles crossing the ankle of people with hemophilic arthropathy (PWHA; *n* = 14) at their preferred velocity (red), the control group (*n* = 13) at their preferred velocity (CG-pref, black), and during the slow velocity condition (CG-slow, blue). Data are plotted as a function of normalized step time (0, heel strike) and expressed as mean with 95% confidence interval for CG. Vertical dashed lines indicate transition from stance to swing phase (black, CG-pref; blue, CG-slow; red, PWHA). **(Bottom two rows)** Time-dependent *t* values of the statistical parametric mapping (SPM) for groups comparison. Horizontal red dashed line indicates *p* = 0.05 level. Gray zones indicate regions with statistically significant differences. a.u, Arbitrary unit.

**FIGURE 6:**
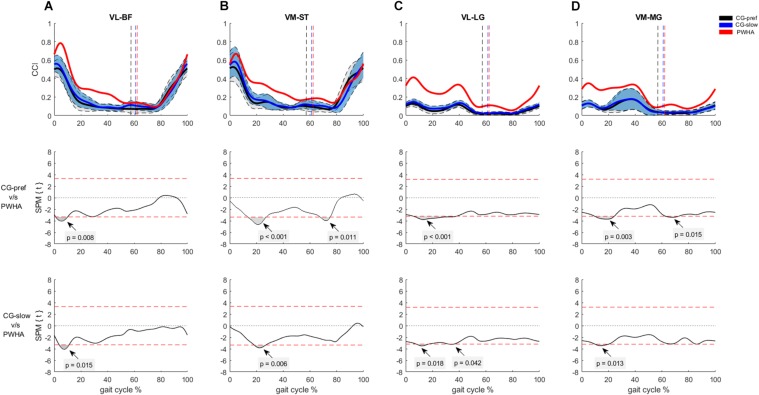
Co-contraction of muscles crossing the ankle during gait. **(A)** Con-contraction index (CCI) between vastus lateralis and biceps femoris (VL-BF). **(B)** CCI between vastus medialis and semitendinosus (VM-ST). **(C)** CCI between vastus lateralis and lateral gastrocnemius (VL-LG). **(D)** CCI between vastus medialis and medial gastrocnemius (VM-ST). **(Top row)** CCI of muscles crossing the knee of people with hemophilic arthropathy (PWHA; *n* = 14) at their preferred velocity (red), the control group (*n* = 13) at their preferred velocity (CG-pref, black), and during the slow velocity condition (CG-slow, blue). Data are plotted as a function of normalized step time (0, heel strike) and expressed as mean with 95% confidence interval for CG. Vertical dashed lines indicate transition from stance to swing phase (black, CG-pref; blue, CG-slow; red, PWHA). **(Bottom two rows)** Time-dependent *t* values of the statistical parametric mapping (SPM) for groups comparison. Horizontal red dashed line indicates *p* = 0.05 level. Gray zones indicate regions with statistically significant differences. a.u, Arbitrary unit.

For the co-contraction of pair muscles crossing the ankle, PWHA showed higher CCI during the stance phase for MG-TA (at ∼20% of the step cycle; *p* = 0.010, *d* = 1.34) and LG-TA (at ∼20% of the step cycle; *p* < 0.001, *d* = 1.46) and during the swing phase for MG-TA (at ∼70% of the step cycle; *p* = 0.032, *d* = 1.32) and LG-TA (at ∼60% of the step cycle; *p* = 0.016, *d* = 1.06) (Figure 5). No difference between groups was found for SOL-TA (Figure 5). For the co-contraction of pair muscles crossing the knee, PWHA showed a higher CCI during the stance phase for VL-BF (at ∼10% of the step cycle; *p* = 0.008, *d* = 1.46), VM-ST (at ∼20% of the step cycle; *p* < 0.001, *d* = 1.58), VL-LG (at ∼20% of the step cycle; *p* < 0.001, *d* = 1.34), VM-MG (at ∼20% of the step cycle; *p* = 0.003, *d* = 1.40), and during the swing phase for VM-ST (at ∼70% of the step cycle; *p* = 0.011, *d* = 1.53) and VM-MG (at ∼70% of the step cycle; *p* = 0.015, *d* = 1.36) (Figure 6).

Comparing muscle activity patterns between groups at the same velocity revealed different activity patterns in the plantar flexors and knee flexors. In the muscles crossing the ankle, PWHA showed relatively higher activation of LG during the stance phase (at ∼10–20% of the gait cycle; *p* = 0.002, *d* = 1.46) (Figure 2). In the muscles crossing the knee, PWHA showed relatively higher activation of BF during the stance phase (at ∼10% of the step cycle; *p* = 0.007, *d* = 1.44) (Figure 3). For the muscles crossing the hip, no differences between group were found for GMAX and GMED (Figure 4).

For the co-contraction of pair muscles crossing the ankle, PWHA showed a higher CCI during the stance phase for MG-TA (at ∼20% of the step cycle; *p* = 0.005, *d* = 1.33) and LG-TA (at ∼20% of the step cycle; *p* < 0.001, *d* = 1.41) and during the swing phase for MG-TA (at ∼60% of the step cycle; *p* = 0.047, *d* = 0.918) and LG-TA (at ∼60% of the step cycle; *p* = 0.013, *d* = 1.24) (Figure 5). No difference between group was found for the pair SOL-TA (Figure 5). For the co-contraction of pair muscles crossing the knee, PWHA showed a higher CCI during the stance phase for VL-BF (at ∼10% of the step cycle; *p* = 0.015, *d* = 1.47), VM-ST (at ∼20% of the step cycle; *p* = 0.006, *d* = 1.41), VL-LG (at ∼10% of the step cycle; *p* = 0.018, *d* = 1.30; at ∼40% of the step cycle; *p* = 0.042, *d* = 1.29), and VM-MG (at ∼20% of the step cycle; *p* = 0.013, *d* = 1.30) (Figure 6).

### Timing and Duration of EMG Activity Between Groups

**FIGURE 7:**
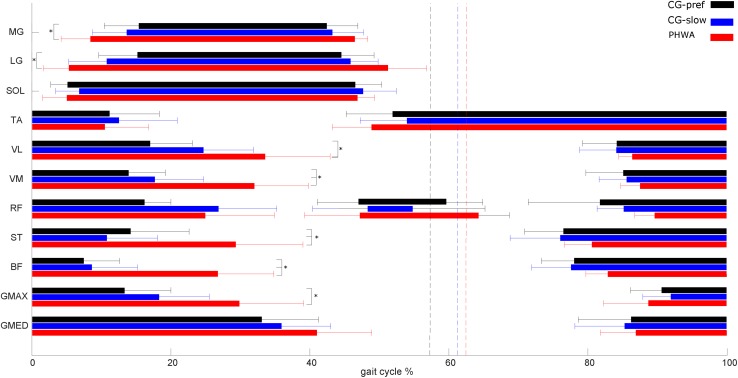
Electromyography (EMG) burst on- and offset during gait. EMG burst on- and offset during gait of people with hemophilic arthropathy (PWHA; *n* = 14) at their preferred velocity (red), the control group (*n* = 13) at their preferred velocity (CG-pref, black), and during the slow velocity condition (CG-slow, blue). The second burst of rectus femoris muscle during stance to swing transition was present in only part of the subjects: 11/13 for the CG-pref, 6/13 for CG-slow, and 9/14 in PWHA. Data are plotted as a function of normalized step time (0, heel strike) and expressed as mean with 95% confidence interval for CG. Vertical dashed lines indicate transition from stance to swing phase (black, CG-pref; blue, CG-slow; red, PWHA). Medial gastrocnemius (MG), lateral gastrocnemius (LG), soleus (SOL), tibialis anterior (TA), vastus lateralis (VL), vastus medialis (VM), rectus femoris (RF), semitendinosus (ST), biceps femoris (BF), gluteus maximus (GMAX), and gluteus medius (GMED). Data are expressed as mean and 95% confidence interval.

Comparing the onset/offset activation pattern of individual muscles between groups at their preferred velocity revealed different timing and duration in EMG activity in various muscles. In the muscles crossing the ankle, the MG and LG muscles in PWHA showed an earlier onset during the stance phase and longer total duration of activity (Table 3 and Figure 7). In the muscles crossing the knee, PWHA showed a later offset during the stance phase of VM, VL, ST, and BF muscles and the longer total duration of activity of VM, VL, and BF muscles (Table 3 and Figure 7). In the muscles crossing the hip, the GMAX muscle showed a later offset during the stance phase and a longer total duration of activity (Table 3 and Figure 7).

**TABLE 3:** Statistical results (*p* values) and effect size (ES) for EMG burst onset–offset and burst duration.

	**Muscle**	**Variable**	**PWHA vs. CG-pref**	**PWHA vs. CG-slow**
			***p* value (*d*)**	***p* value (*d*)**
Muscles crossingthe ankle	Medial gastrocnemius	Onset	**0.034 (0.91)**	0.103 (0.69)
		Offset	0.198 (0.73)	0.610 (0.59)
		Duration	**0.001 (1.53)**	**0.005 (1.21)**
	Lateral gastrocnemius	Onset	**0.006 (1.30)**	0.058 (0.72)
		Offset	0.061(0.76)	0.107 (0.64)
		Duration	**<0.001 (1.54)**	**0.007 (1.09)**
	Soleus	Onset	0.636 (0.05)	0.269 (0.32)
		Offset	0.942 (0.08)	0.680 (0.11)
		Duration	0.645 (0.13)	0.396 (0.22)
	Tibialis anterior	Onset	0.198 (0.32)	0.089 (0.49)
		Offset	0.899 (0.05)	0.649 (0.18)
		Duration	0.396 (0.23)	0.437 (0.22)
Muscles crossing the knee	Vastus lateralis	Onset	0.752 (0.38)	0.627 (0.37)
		Offset	**0.011 (1.21)**	0.167 (0.60)
		Duration	**0.027 (1.05)**	0.369 (0.41)
	Vastus medialis	Onset	0.752 (0.35)	0.512 (0.36)
		Offset	**0.002 (1.53)**	**0.014 (1.11)**
		Duration	**0.005 (1.30)**	**0.020 (0.96)**
	Rectus femoris	Onset	0.216 (0.64)	0.145 (0.80)
		Offset	0.275 (0.67)	0.577 (0.12)
		Onset (second burst)	0.209 (0.60)	0.054 (1.19)
		Offset (second burst)	0.998 (0.01)	0.840 (0.14)
		Duration	0.716 (0.06)	0.790 (0.08)
	Semitendinosus	Onset	0.396 (0.53)	0.528 (0.49)
		Offset	**0.031 (0.97)**	**0.003 (1.25)**
		Duration	0.069 (0.61)	**0.035 (0.75)**
	Biceps femoris	Onset	0.152 (0.75)	0.065 (0.72)
		Offset	**0.001 (1.64)**	**0.002 (1.42)**
		Duration	**0.023 (1.08)**	0.077 (0.81)
Muscles crossing the hip	Gluteus maximus	Onset	0.647 (0.18)	0.409 (0.32)
		Offset	**0.003 (1.17)**	0.055 (0.80)
		Duration	**0.003 (1.22)**	0.073 (0.91)
	Gluteus medius	Onset	0.790 (0.08)	0.903 (0.16)
		Offset	0.369 (0.58)	0.577 (0.40)
		Duration	0.409 (0.40)	0.610 (0.20)

*Comparison between the control group during preferred (CG−pref; *n* = 13) and slow walking velocity (CG−slow; *n* = 13) and people with hemophilic arthropathy (PWHA; *n* = 14) during preferred walking velocity. The second burst of rectus femoris muscle during stance to swing transition was present in only part of the subjects: 11/13 for the CG-pref, 6/13 for CG-slow, and 9/14 in PWHA. Effect size (*d*) Significant differences (*p* < 0.05) are in bold.*

Differences in timing and duration of EMG activity were also found when comparing the onset/offset activation pattern of individual muscles between groups at the same velocity. In the muscles crossing the ankle, PWHA showed a longer total duration of activity of MG and LG (Table 3). In the muscles crossing the knee, the VM, ST, and BF showed a later offset during the stance phase in PWHA and the longer total duration of activity of VM and ST (Table 3 and Figure 7).

### Kinematic and the Temporal Gait Parameters

**FIGURE 8:**
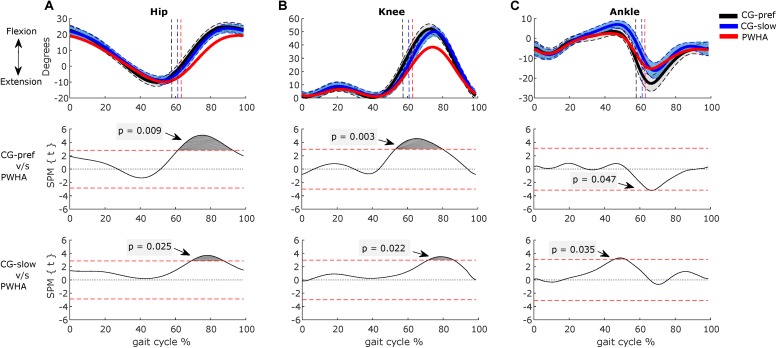
Comparison between groups of kinematics during gait. **(A)** Hip. **(B)** Knee. **(C)** Ankle. **(Top row)** Kinematics of people with hemophilic arthropathy (PWHA; *n* = 14) at their preferred velocity (red), the control group (*n* = 13) at their preferred velocity (CG-pref, black), and during the slow velocity condition (CG-slow, blue). Data are plotted as a function of normalized step time (0, heel strike) and expressed as mean with 95% confidence interval for CG. Vertical dashed lines indicate transition from stance to swing phase (black, CG-pref; blue, CG-slow; red, PWHA). **(Bottom two rows)** Time-dependent *t* values of the statistical parametric mapping (SPM) for groups comparison. Horizontal red dashed line indicates *p* = 0.05 level. Gray zones indicate regions with statistically significant differences. Data are expressed as mean and 95% confidence interval.

Comparing the kinematics between groups at their preferred velocity, the SPM analysis showed significant differences of the hip, knee, and ankle joints (Figure 8). In PWHA, the hip joint showed the lower amplitude of flexion during the swing phase (at around 60–90% of the cycle; *p* = 0.009, *d* = 1.59) and lower ROM (mean difference of 5.6°, *p* = 0.008, *d* = 1.12) (Table 4). The knee joint in PWHA showed a lower amplitude of flexion during the transition from stance to swing phase (at around 53–80% of the cycle; *p* = 0.003, *d* = 1.47) and lower ROM (mean difference of 14.4°, *p* < 0.001, *d* = 1.85) (Table 4). The ankle joint in PWHA showed a lower amplitude of plantar flexion during stance (at around 65–68% of the gait cycle; *p* = 0.047, *d* = 1.22) and lower ROM (mean difference of 7.5°, *p* = 0.015, *d* = 1.31) (Table 4). Regarding temporal gait parameters, the preferred velocity of PWHA was lower than that of the CG (difference, 0.1 m/s; *p* = 0.044, *d* = 0.80). PWHA compared to CG at preferred velocity showed a similar cycle time (difference, 0 s; *p* = 0.480, *d* = 0.28), a higher coefficient of variation of time cycle (difference, 0.9%; *p* = 0.001, *d* = 1.47) and longer stance time (difference, 5.1%; *p* = 0.002, *d* = 1.44) (Table 4).

**TABLE 4:** Temporal variables and total range of motion (ROM) during walking.

**Variables**	**CG-pref**	**CG-slow**	**PWHA**	**PWHA vs. CG-pref**	**PWHA vs. CG-slow**
				***p* value (*d*)**	***p* value (*d*)**
Velocity in 30 m (m/s)	1.2 ± 0.2	1.0 ± 0.2	1.1 ± 0.1	**0.044 (0.80)**	0.203 (0.58)
Time cycle (s)	1.1 ± 0.1	1.2 ± 0.1	1.1 ± 0.1	0.480 (0.28)	**0.010 (1.07)**
CV of time cycle (%)	1.7 ± 0.5	2.0 ± 0.6	2.6 ± 0.7	**0.001 (1.47)**	**0.010 (1.02)**
Stance time (%)	57.3 ± 3.0	61.3 ± 3.6	62.4 ± 3.8	**0.002 (1.44)**	0.544 (0.26)
ROM of hip (°)	35.4 ± 4.9	33.3 ± 5.5	29.8 ± 5.1	**0.008 (1.12)**	0.096 (0.66)
ROM of knee (°)	53.7 ± 7.1	51.2 ± 5.1	39.3 ± 8.3	**<0.001 (1.85)**	**0.002 (1.71)**
ROM of ankle (°)	27.3 ± 5.8	25.7 ± 4.4	19.8 ± 5.7	**0.015 (1.31)**	**0.006 (1.16)**

*Comparisons between the control group during preferred (CG-pref; *n* = 13) and slow walking velocity (CG-slow; *n* = 13) and people with hemophilic arthropathy (PWHA; *n* = 14) during preferred walking velocity. Coefficient variation (CV). Effect size (*d*). Data are expressed as mean and standard deviation. Significant differences (*p* < 0.05) are in bold.*

Comparing the kinematics between groups at the same velocity, the SPM analysis showed a significant difference in hip, knee, and ankle angles (Figure 8). In PWHA, the hip joint showed the lower amplitude of ROM during the swing phase (at around 69–88% of the cycle; *p* = 0.025, *d* = 1.27); however, no difference between PWHA and controls was found in ROM of hip (Table 4). The knee joint in PWHA showed a lower amplitude of ROM during the swing phase (at around 72–87% of the cycle; *p* = 0.022, *d* = 1.27) and a lower ROM (difference of 11.9°, *p* = 0.002, *d* = 1.71) (Table 4). The ankle joint in PWHA showed a lower amplitude of dorsi-flexion during the stance phase (at around 45–52% of gait cycle *p* = 0.035, *d* = 1.24) and a lower ROM (difference of 5.9°, *p* = 0.006, *d* = 1.16) (Table 4). The preferred velocity of PWHA was similar than the slow velocity of CG (difference, 0.1 m/s; *p* = 0.203, *d* = 0.58). PWHA compared to CG at the same velocity showed a lower cycle time (difference, 0.1 s; *p* = 0.010, *d* = 1.07), a higher coefficient of variation of cycle time (difference, 0.6%; *p* = 0.010, *d* = 1.02) and a similar stance time (difference, 1.1%; *p* = 0.544, *d* = 0.26) (Table 4). These results indicate that the kinematics of the leg joints in PWHA differs from that of the CG.

## Discussion

The aim of the present study was to assess if the neural control of individual muscles, coordination between antagonistic muscle pairs, and joint kinematics during gait are affected in PWHA. The main results of our study are that PWHA differs from controls with regard to the following: (i) EMG amplitudes of the triceps surae and hamstrings muscles, (ii) level of co-contraction for several antagonistic muscles crossing the ankle and knee (i.e., MG-TA, LG-TA, VL-BF, VM-ST, LG-VL, and MG-VM), (iii) timing and duration of EMG activity of several muscles (i.e., MG, LG, VL, VM, ST, BF, and GMAX), and (iv) range of motion of in the ankle, knee, and hip joints. To the authors’ current knowledge, this study is the first to report that the muscle activity and antagonistic co-contraction patterns and the temporal on–off activation pattern of several leg muscles during gait in PWHA differ from those in CG.

### Differences in Neuromuscular Control

In the muscles crossing the ankle, the LG of PWHA showed relatively higher activation during the first half of the stance phase and an earlier onset for MG and LG. The different activation pattern of LG (and to a lesser extent also MG) observed in PWHA is similar to that reported for people with an elongated Achilles tendon following surgical reconstruction (Suydam et al., 2015) and that for people with ankle OA (Doets et al., 2007). The earlier activation of MG and LG muscles and increased amplitude during the first half of the stance phase make the activation pattern similar to that of SOL muscle (Figures 2, 7). One explanation of this finding could be the decreased stiffness of Achilles tendon with severe ankle arthropathy in PWHA (Cruz-Montecinos et al., 2019b), affecting muscle force transmission to the calcaneus. This neural adaptation may be a compensatory strategy to maintain muscle fascicles work at about constant length (Ishikawa et al., 2005; Lichtwark and Wilson, 2006), causing an extra stretch of the Achilles tendon (Suydam et al., 2015). A second explanation for the altered neuromuscular control may be the newly formed connective tissues (i.e., scar tissue) between SOL and gastrocnemius (GA), as a result of repetitive intra-articular and intra-/intermuscular bleedings in PWHA (Ribbans and Rees, 1999; Melchiorre et al., 2017). In a recent study on rats, a shift from preferential recruitment of SOL muscle to preferential recruitment of GA muscle during locomotion in response to increased stiffness of intermuscular connective tissues was reported (Bernabei et al., 2017). To assess this for our data, we calculated the slope ratio between SOL/LG EMG during the first half of the stance phase at the same gait velocity (Bernabei et al., 2017). The slope ratio of PWHA was lower (53.6% less, *p* = 0.028, *d* = 0.90) than that of CG, indicating a relative shift toward preferential recruitment of LG. These results are in agreement with adaptations in response to enhanced connectivity between SOL and LG (Bernabei et al., 2017). However, future studies are needed to confirm changes in the mechanical coupling between ankle plantar flexors in PWHA.

We found a relatively lower activity (∼20% less) of SOL muscle observed during the push-off phase at 40% of the gait cycle in PWHA (Figure 2). A lower amplitude of SOL activity during the stance phase has also been reported in people with ankle arthrodesis (Wu et al., 2000). The relatively lower activity of SOL muscle at 40% of the gait cycle observed in PWHA may be explained by a lower muscle force (Sinkjær et al., 2000; Af Klint et al., 2010), reducing force feedback, and less dorsi-flexion of the ankle joint (Figures 2, 8), reducing length feedback.

The early onset of GA activity and the resulting greater overlap with TA activity during the stance phase (Figure 7), as well as the higher co-contraction between MG-TA and LG-TA observed in PWHA, indicate altered coordination between antagonistic muscles. In PWHA, increased co-contraction between antagonistic muscles (i.e., TA, MG, and LG) has been reported only during a static task (i.e., upright posture), which appears to compensate for their joint damage (Kurz et al., 2012, 2019). The increased co-contraction between MG-TA and LG-TA during stance, as found here, has also been reported in people with ankle OA (Doets et al., 2007; Von Tscharner and Valderrabano, 2010). This may be a strategy to increase the stability of the ankle joint during reception and push-off action, also limiting the load exerted on the ligaments and cartilage tissue (O’Connor, 1993). Note, however, that co-contraction increases the intraarticular load (Trepczynski et al., 2018), which may accelerate the progression of cartilage degeneration (Griffin and Guilak, 2005; Richards and Higginson, 2010; Knarr et al., 2012).

In the muscles crossing the knee, we observed relatively higher amplitudes of activation and later offset of knee flexors and extensors during the stance phase in PWHA. Several studies have been reported that longer and relatively higher activation of knee flexors in people with knee OA contributes to joint degeneration (Hubley-Kozey et al., 2009; Hodges et al., 2016; Trepczynski et al., 2018). Different mechanisms have been proposed to explain the longer activation of knee flexors during gait in knee OA. The laxity of the knee joint could contribute to adopting a more prolonged activity of knee flexors during the acceptance phase facilitating joint stability (Lewek et al., 2004; Hubley-Kozey et al., 2008). In addition, similar to the Achilles tendon the mechanical properties of the patellar tendon may be more compliant in PWHA, contributing to a later offset of VM and VL.

The later offset in knee flexors and the resulting greater overlap with activity of knee extensors during the stance phase (Figure 7), as well as higher co-contraction between VL-BF, VM-ST, LG-VL, and MG-VM observed in PWHA, indicate altered coordination between antagonistic muscles. The higher co-contraction between knee flexor and extensor muscles has been reported in people with knee OA (Hubley-Kozey et al., 2009; Alnahdi et al., 2012; Hodges et al., 2016; Preece et al., 2016). Quadriceps strength is strongly and inversely correlated with muscle co-contraction in both healthy people and people with knees with articular cartilage defects (Thoma et al., 2016). In people with knee OA and PWHA, a reduced quadriceps strength has been reported (Hilberg et al., 2001; González et al., 2007; Alnahdi et al., 2012). These results suggest that the higher co-contraction between muscles crossing the knee may serve to stabilize the joint similar to that reported for people with knee OA (Smith et al., 2019). In PWHA, we also observed that the co-contraction between GA and knee extensors during the stance phase is increased. Similar results have been reported in people with knee OA (Lewek et al., 2005). Owing to the biarticular function of GA (i.e., plantar flexion and knee flexion), the higher co-contraction observed between GA and knee extensors in PWHA may be an adaptation to increase the stability of the knee during the stance phase, but this may also have consequences for its function at the ankle joint.

In addition to the above described factors (e.g., muscle strength, joint stability), pain has been related to changes in neuromuscular control during gait in knee OA (Hortobágyi et al., 2005). This has received little attention in PWHA (Cruz-Montecinos et al., 2019a). The intensity of pain during gait reported in this study was mild (median of Visual Analogue Scale 1; min, 0; max, 5 points, Table 1). Therefore, we do not expect that pain plays an important role in our results. However, futures studies are needing to assess if and in what way the intensity of pain and duration of arthropathy symptoms affects neuromuscular control during gait and more challenging activities (i.e., stair negotiation) (Smith et al., 2019).

The present results may help to design new physical therapy approaches to improve the neuromuscular control during gait in PWHA. In knee OA, for instance, it has been found that a neuromuscular re-education (Preece et al., 2016) and exercise program integrated with self-management education reduced the co-contraction between knee extensors and flexors during gait (Al-Khlaifat et al., 2016). However, future studies are needed to probe if these approaches mentioned above have the same effects in PWHA.

### Changes in Leg Kinematics

We found in PWHA a reduced sagittal plane ROM in the ankle, knee, and hip. The reduced ankle ROM in PWHA compared to controls during preferred and slow velocity (mean difference, 6.4 and 4.8°, respectively), was similar than reported in previous studies in people with ankle OA and hemophilic ankle arthropathy (Lobet et al., 2010; Nüesch et al., 2014). In addition, PWHA showed a lower amplitude of plantar flexion compared to the CG during preferred velocity and lower amplitude of dorsi-flexion for the ankle joint compared to the CG during slow velocity. The lower amplitude of plantar flexion could be explained by the altered co-contraction and lower stiffness of the Achilles tendon. The decreased plantar flexion observed in PWHA may be explained by the increased co-contraction between GA and TA and reduced SOL activity during push-off, similarly to that reported in elderly (Franz and Kram, 2013). The lower stiffness of the Achilles tendon reported in PWHA (Cruz-Montecinos et al., 2019b) and elderly (Delabastita et al., 2018), affecting force transmission from triceps surae muscles to the calcaneus (Don et al., 2007), maybe responsible for less ankle plantar flexion. The decreased dorsi-flexion in PWHA may be explained by articular and non-articular factors. Different surgical approaches improve dorsi-flexion (i.e., increased passive ROM) in PWHA, such as the release of the posterior joint capsule (Barg et al., 2016), the anterior osteophyte on the tibiotalar joint (Yoo et al., 2019), and Achilles tendon lengthening (Ribbans and Rees, 1999).

The PWHA recruited in our study had a passive ROM higher than 60° in the knee. Despite that, in PWHA, a smaller knee flexion angle and lower ROM were observed compared with CG for both velocities. In knee OA, several studies have reported the impact of a limited knee ROM on gait (Al-Zahrani and Bakheit, 2002; Gök et al., 2002; Astephen et al., 2008). The limited knee ROM compared to CG during preferred and slow velocity (mean difference, 14 and 12°, respectively) was similar to that reported in previous studies in people with knee OA (Gök et al., 2002; Astephen et al., 2008). One explanation of this finding could be a lower knee flexion velocity at toe-off, resulting in a lower peak knee flexion during the swing phase (Piazza and Delp, 1996; Goldberg et al., 2003, 2004). In addition, in the present study, knee flexion velocity at toe-off was lower in PWHA (27%, *p* < 0.001) than that of CG (data not shown).

In the hip joint, we observed a lower ROM in PWHA comparing to CG at the preferred velocity and lower flexion angle during the swing phase in PWHA comparing to CG at both velocities. It is not common in PWHA that the hip is affected by recurrent bleeding, and the hip is generally affected at a later stage (Carulli et al., 2017). Therefore, the lower hip flexion angle during the swing phase may be (partly) explained by changes of knee and ankle muscle activity and the reduced step length.

### Limitations

This study has some limitations. First, joint kinetics were not assessed. Therefore, it is unclear if loading of the joints, and hence the mechanical demands, were different in PWHA. Second, the applied EMG normalization method, the maximum value of all included steps, limits the interpretation of differences in intensity between groups (Benoit et al., 2003; French et al., 2015). We selected this method, and not one using EMG during maximal voluntary contraction, because the latter cannot be recorded reliably due to the potential provocation of pain incrementing the intersubject variability (Cronin et al., 2015). Third, due to the greater variability of gait observed in PWHA, an outlier may reduce the normalized EMG values for the other cycles and, thus, affect the mean. However, using the median instead of the maximum, as proposed earlier (Cheung et al., 2009), did yield similar results. Four, we used IMU sensors to assess only the sagittal plane kinematics of hip, knee, and ankle joints. The other planes (i.e., internal–external rotation and abduction–adduction) were not assessed because the accuracy is inadequate (Zhang et al., 2013). The IMU is less sensitive to detect changes than the traditional camera-based optical motion capture systems (Cooper et al., 2009). Despite that, in our study, we observed significant differences between PWHA and CG in ankle, knee, and hip joint kinematics. In addition, the kinematics of the CG were similar to those reported for healthy people during overground walking assessed with traditional camera-based optical motion capture systems (Fukuchi et al., 2018) and IMU sensor in knee OA (Chapman et al., 2019). Five, to exclude joint disease in the CG, we used the HJHS and clinical criteria (i.e., Alt-man’s criteria) based on previous studies in knee OA (Altman et al., 1986; Na and Buchanan, 2019). It would have been better to confirm the inexistence of asymptomatic joint disease for the CG using radiological scores by magnetic resonance imaging or ultrasound (Lundin et al., 2012; De La Corte-Rodriguez et al., 2018).

## Conclusion

In conclusion, the neural control of individual muscles and coordination between antagonistic muscles during gait in PWHA differs substantially from control subjects. To explain the changes in neuromuscular control in PWHA, future studies should focus on the potential mechanisms, their interaction with joint damage, and possibly pain.

## Data Availability Statement

The datasets analyzed in this manuscript are available in the article/Supplementary Material.

## Ethics Statement

The studies involving human participants were reviewed and approved by the Northern Metropolitan Health Service of Santiago, Chile. The patients/participants provided their written informed consent to participate in this study.

## Author Contributions

CC-M, SP-A, MC, and HM conceived the project idea. CC-M conducted the experiments. CC-M and HM conducted the data analyses and drafted the manuscript. SP-A, MC, and FQ contributed to revising the manuscript.

## Conflict of Interest

The authors declare that the research was conducted in the absence of any commercial or financial relationships that could be construed as a potential conflict of interest.

## Footnotes

1 http://www.spm1d.org/index.html
