# Supplementary material for: Changes in Muscle Activity Patterns and Joint Kinematics During Gait in Hemophilic Arthropathy
Source: Front Physiol. 2020 Jan 31;10:1575. doi: 10.3389/fphys.2019.01575 (PMC7006441; doi:10.3389/fphys.2019.01575)
Supplement: Supplementary file 1 [file Data_Sheet_1.zip › Data Sheet 1/Captions.docx]

**Captions for Supplementary Datasheet**

1.-Clinical data (*Excel file*). Data supporting the results of table 1 and table 2.

2.-ON-OFF dataset (*Excel file*). Data supporting the results of table 3 and figure 7.

3.-EMG_MEAN_FRONTIERS (*Matlab file*). Envelope EMG signals in a 3D matrix (1500 points x 11 muscles x subjects) and description of muscle channels. Data supporting the results of figure 2 to figure 6.

4.-TEMPORAL_VARIABLES dataset (*Excel file*). Data supporting the results of table 4.

5.-Kinematics for the hip, knee, and ankle (*Matlab file*). Data supporting the results of table 4 and figure 8.
